# Preoperative Physical Fitness and Short-Term Postoperative Outcomes in Patients Undergoing Elective Cardiac Surgery: A Retrospective Cohort Study

**DOI:** 10.1177/10892532261427827

**Published:** 2026-02-21

**Authors:** Zoe van der Hoofd, Hanneke C. van Dijk-Huisman, Bart C. Bongers, Bart Scheenstra, Jos Maessen, Antoine F. Lenssen

**Affiliations:** 1 Department of Physical Therapy, Maastricht University Medical Center+, Maastricht, The Netherlands; 2 Department of Epidemiology, Care and Public Health Research Institute (CAPHRI), Faculty of Health Medicine and Life Sciences, 5211 Maastricht University, Maastricht, The Netherlands; 3 Department of Nutrition and Movement Sciences, Institute of Nutrition and Translational Research in Metabolism (NUTRIM), Faculty of Health Medicine and Life Sciences, 5211 Maastricht University, Maastricht, The Netherlands; 4 Department of Surgery, Institute of Nutrition and Translational Research in Metabolism (NUTRIM), Faculty of Health Medicine and Life Sciences, 5211 Maastricht University, Maastricht, The Netherlands; 5 Department of Cardiac Rehabilitation, 72480 Basalt, Leiden, The Netherlands; 6 Department of Cardiothoracic Surgery, Heart and Vascular Center, 5211 Maastricht University Medical Center, Maastricht, The Netherlands; 7 Department of Cardiothoracic Surgery, Cardiovascular Research Institute Maastricht (CARIM), 5211 Maastricht University, Maastricht, The Netherlands

**Keywords:** thoracic surgery, risk assessment, cardiorespiratory fitness, perioperative care, postoperative complications, prehabilitation

## Abstract

Preoperative cardiorespiratory fitness, muscle strength, and frailty influence outcomes after cardiac surgery, but these modifiable physical factors are often not routinely incorporated into standardized risk assessments. This study examined the association between preoperative physical fitness and adverse postoperative outcomes across different elective cardiac surgery procedures. Logistic regression analyses were used to assess the association between preoperative cardiorespiratory fitness, muscle strength, functional mobility, frailty, and quality of life and delayed postoperative recovery of physical functioning (Modified Iowa Level of Assistance Scale), in-hospital complications, and postoperative atrial fibrillation in patients undergoing cardiac surgery via sternotomy, mini-thoracotomy, and transfemoral incision (transcatheter aortic valve implantation). Results showed that higher patient-reported preoperative cardiorespiratory fitness, functional mobility, frailty, and physical health-related quality of life were significantly associated with faster recovery of physical functioning and fewer postoperative complications in patients undergoing sternotomy. In patients undergoing mini-thoracotomy, preoperative cardiorespiratory fitness and functional mobility were significantly associated with in-hospital complications. No significant associations were found in patients undergoing transfemoral incision (transcatheter aortic valve implantation). Conclusively, preoperative physical fitness is associated with postoperative outcomes in patients undergoing sternotomy. These findings highlight the importance of incorporating physical fitness assessments into standard preoperative care to facilitate preoperative shared decision-making and optimize modifiable preoperative risk factors.

## Graphical abstract

**Graphical Abstract:**
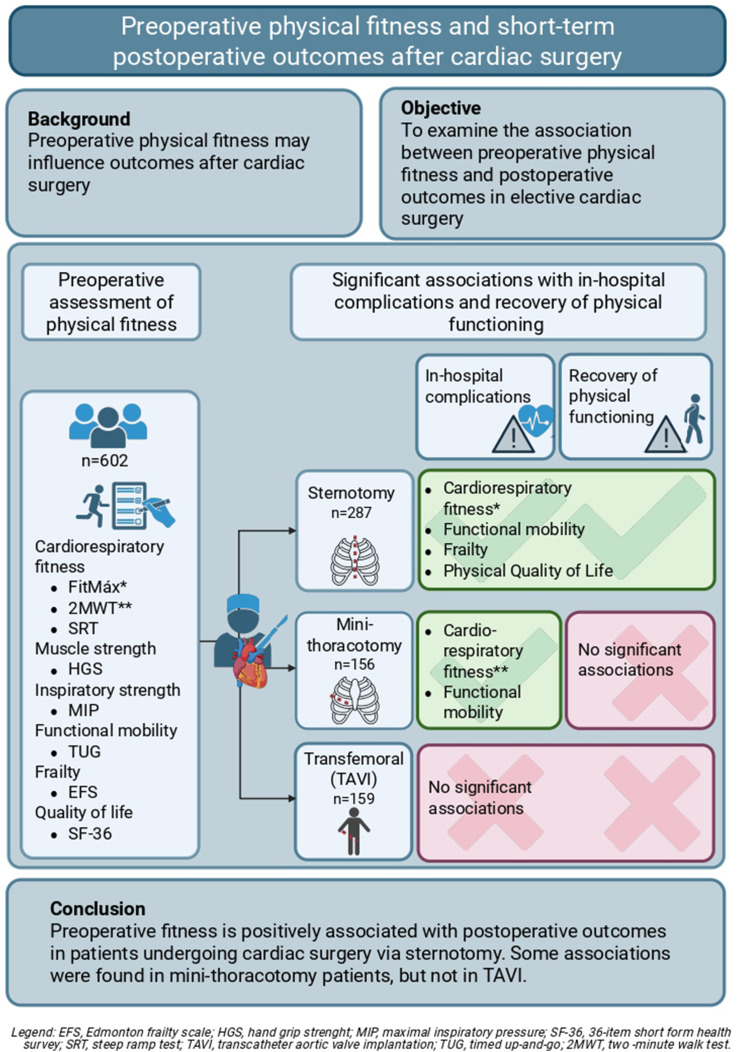

## Background

Cardiac surgery plays a crucial role in managing cardiovascular disease and significantly improves survival and quality of life.^1^ However, these complex procedures carry a substantial risk of adverse short-term postoperative outcomes.^2^ Modifiable risk factors, such as low cardiorespiratory fitness,^3,4^ low muscle strength,^5^ and low inspiratory muscle strength,^6^ have been associated with prolonged length of hospital stay, increased complications, and higher mortality. However, these associations are not fully understood, as studies report inconsistent findings and methodological limitations.^3-6^

Preoperative risk assessments for cardiac surgery, such as the Society of Thoracic Surgeons (STS) score^7^ and the European System for Cardiac Operative Risk Evaluation (EuroSCORE),^8^ are developed to estimate perioperative mortality and are also associated with postoperative complications and hospital length of stay.^9^ While valuable, these assessments primarily consider demographic and clinical variables, but often do not incorporate modifiable factors such as preoperative physical fitness. Including these modifiable factors could increase the predictive value of the preoperative assessment and may create opportunities to optimize preoperative status^10^ and improve patient- and treatment-related outcomes.

Despite growing evidence supporting the role of modifiable physical fitness in preoperative risk assessment, there is no consensus on the optimal performance tests or questionnaires. Existing studies often focus on specific procedures, such as sternotomy or transfemoral aortic valve implantation (TAVI), limiting generalizability across cardiac surgery types.^3,4,11^ Moreover, prior studies frequently rely on institutionally biased outcomes, such as length of stay, rather than objective, patient-centered measures of functional recovery. This highlights the need for a broader understanding of which aspects of preoperative physical fitness influence postoperative outcomes, particularly in increasingly heterogeneous patient populations and surgical techniques. Greater insight could facilitate preoperative decision-making and provide more targeted prehabilitation interventions.^12^

This study aimed to investigate the association between preoperative physical fitness and delayed recovery of functional functioning, in-hospital complications and postoperative atrial fibrillation in patients undergoing elective cardiac surgery, with stratification based on surgical invasiveness (sternotomy, mini-thoracotomy, and transfemoral incision).

## Methods

This single-center retrospective observational cohort study was conducted in a tertiary referral hospital for cardiac surgery. It followed the principles of the Declaration of Helsinki and received approval by the Medical Ethical Committee of Maastricht University Medical Centre/Maastricht University (METC azM/UM, reference 2023-0372). Data were collected from April 2023 to August 2024.

### Study Population

Patients scheduled for elective cardiac surgery underwent preoperative risk assessment by a multidisciplinary team, including a physician assistant, cardiothoracic resident, surgeon, anesthesiologist, and physical therapist. The time between assessment and surgery varied from weeks to months.

Eligible patients were ≥18 years, had a preoperative assessment ≤180 days before surgery, consented to data use, and undergoing elective surgery (e.g., coronary revascularization, valve repair/replacement, aortic aneurysm repair, or combined procedures). Patients undergoing stand-alone hybrid atrial fibrillation ablation were excluded. Surgical procedures included sternotomy, mini-thoracotomy, and transfemoral incision (e.g., TAVI).

### Preoperative Risk Assessment of Physical Fitness

Physical fitness was evaluated preoperatively by a physical therapist as part of routine care.

#### Cardiorespiratory Fitness

Cardiorespiratory fitness was assessed using the steep ramp test (SRT), a short-term maximal exercise test on a calibrated cycle ergometer (eBike III comfort, GE Healthcare),^13^ following hospital protocols based on the Dutch Cardiology Association’s cardiopulmonary exercise testing protocol (NVVC). It was conducted under continuous electrocardiogram (ECG) monitoring. Patients were instructed to cycle until exhaustion or until the onset of cardiac symptoms, that is, angina pectoris or dizziness. After a 3-minute warm-up at 25 W, workload increased by 25 W every 10 seconds. Patients maintained a pedaling frequency of 60-70 rotations per minute. The test ended when pedaling fell below 60 rotations/min despite encouragement. Peak work rate (WR_peak_) adjusted for body mass (in W/kg) was recorded.^14^ Blood pressure, oxygen saturation, and perceived exertion (modified Borg scale, 6–20) were monitored before, during, and after the test.

Contraindications for the SRT included patients scheduled for aortic aneurysm repair, active endocarditis, aortic dissection, unstable angina pectoris, and patients experiencing previous (pre)syncope, and other. Additionally, the SRT was not performed in absence of a qualified professional for ECG reading. For these patients, the 2-minute walk test (2MWT) was used as an alternative.^15^ Patients walked as fast as possible (without running) for 2 minutes on a 20-meter course. Walking aids were permitted. Distance walked (m), heart rate, oxygen saturation, and perceived exertion were measured before and after. The test was stopped if cardiac symptoms occurred.^15,16^

Patient-reported cardiorespiratory fitness (oxygen uptake at peak exercise (VO_2peak_) was estimated using the FitMáx questionnaire, which consists of three multiple-choice questions evaluating maximum capacity for walking, stair climbing, and cycling. VO_2peak_ was calculated based on the weighted FitMáx score, combined with sex, age, and BMI.^17^

#### General Muscle Strength

Handgrip strength (HGS) (kPa) was measured using the Martin Vigorimeter (Gebrüder Martin, Tuttlingen, Germany). Patients were seated with their shoulder positioned in adduction, elbow positioned in 90° flexion, and forearm and wrist in neutral position. Female patients used a medium-sized balloon (5 cm diameter) and male patients a large-sized balloon (6 cm diameter). Three attempts were performed with the dominant hand, and the highest value was recorded.^18^

#### Inspiratory Muscle Strength

Maximal inspiratory pressure (MIP) (cm H_2_O) was measured using the Micro Respiratory Pressure Meter (Micro Medical Limited, Rochester, United Kingdom) following American Thoracic Society/European Respiratory Society (ATS/ERS) respiratory muscle testing guidelines. Seated patients were exhaled fully, sealed their lips around the mouthpiece, and inhaled as forcefully as possible. The highest value from three attempts with less than 20% variation was recorded.^19^

#### Functional Mobility

Functional mobility was assessed using the timed up-and-go (TUG) test. Patients rose from a standardized armchair, walked a 3-meter course, turned, returned to the chair, and sat down. The time needed to complete the task was recorded with a handheld stopwatch. The fastest time (s) from three attempts was recorded.^20^

#### Frailty

The Edmonton Frailty Scale (EFS) evaluates frailty across nine domains: cognition, general health status, functional independence, social support, medication use, nutrition, mood, continence, and functional performance. The total score (0–17, with higher scores indicating increased frailty) were dichotomized in non-frail (<6) and frail (≥6), using the validated thresholds.^21,22^

#### Quality of Life

Health-related quality of life (QoL) was assessed using the 36-item Short Form Health Survey (SF-36) survey, covering eight dimensions: physical functioning, role limitations (physical and emotional), pain, vitality, general health perception, social functioning, and mental health. A summary score (0–100) reflected overall physical and mental health-related QoL, with higher scores indicating better health.^23^

### Postoperative Data

#### Time to Recovery of Physical Functioning

Recovery of physical functioning was assessed during each daily postoperative physical therapy session using the modified Iowa level of assistance scale (mILAS).^24^ The mILAS assesses the level of assistance needed to perform the following daily activities safely: transferring from a supine to seated position and vice versa, transferring from a seated to standing position, walking, and stair climbing. Each activity is scored from 0 (independent) to 6 (not tested for medical or safety reasons). Recovery of physical functioning was achieved when patients could perform all activities independent (mILAS score in all activities of 0). For patients not requiring stairs at home, stair climbing was excluded. The time to an mILAS score of 0 (in days) was recorded.

#### In-hospital complications

In-hospital complications were obtained from the Netherlands Heart Registration (NHR), including myocardial infarction, vascular complications, arm or leg wound issues, pulmonary infections, respiratory insufficiency, prolonged ventilation (>24 hours), intensive care unit (ICU) readmission, cerebrovascular accidents (CVA), renal insufficiency, gastrointestinal complications, delirium, redo-surgery, and mortality.^25^ In-hospital complications were defined as the occurrence of at least one complication during hospitalization (yes/no).

#### Postoperative Atrial Fibrillation

Postoperative new onset of atrial fibrillation or flutter (POAF) was obtained separately from NHR (yes/no),^25^ as a common postoperative complication not typically influencing early functional recovery.

### Data Collection

The following data was retrospectively extracted from electronic patient records (Systems Applications and Products, Walldorf, Germany) (Table 1).

**Table 1.** Data Collection

Preoperative data encompassed independent variables including:
Demographic characteristics (e.g., age and sex)
Health-related characteristics (body mass index (BMI) (kg/m^2^)
Diabetes mellitus (yes/no)
Chronic pulmonary disease (COPD) (yes/no)
Smoking status (never smoked/ current smoker/former smoker)
Left ventricular ejection fraction (<50%/≥50%)
Estimated glomerular filtration rate (eGFR) (mL/min/1.73 m^2^)
EuroScore II
Surgery characteristics (revascularization surgery via sternotomy or via mini-thoracotomy, valve surgery via sternotomy or via mini-thoracotomy, aortic surgery, TAVI via a transfemoral incision, combined surgery via sternotomy, combined surgery via mini-thoracotomy, or other surgeries)
Physical fitness assessed during the preoperative assessment
Postoperative data comprised the following dependent variables:
Time to recovery of physical functioning (days)
In-hospital complications
Postoperative atrial fibrillation

#### Sample Size Calculation

Associations were analyzed and adjusted for predetermined potential confounders, including Euroscore II, age, BMI, type of incision (sternotomy, mini-thoracotomy or transfemoral incision (TAVI)), and sex, which were selected based on clinical relevance, and previous research. Based on the 10-events-per-variable rule for logistic regression,^26^ with six predictors and an expected 30% complication rate,^27^ a minimum of 200 patients was required.

#### Statistical Analysis

Statistical analyses were performed using the Statistical Package for the Social Sciences (SPSS) for Windows (version 28.0; IBM, SPSS Inc., Chicago, IL, USA).

Descriptive statistics were presented as means ± standard deviations (SD) for normally distributed variables, as medians and interquartile ranges (IQR) for non-normally distributed variables, and as numbers and percentages for categorical variables. Normality of continuous variables was assessed using the Kolmogorov–Smirnov test. Categorical variables were compared using Fisher’s exact test or the Chi-square test, as appropriate. For continuous variables, the independent sample t-test was applied to normally distributed data, while the Mann–Whitney U test was used for skewed data.

Surgical procedures were compared using ANOVA with Bonferroni correction for multiple comparisons. When Levene’s test indicated unequal variances, the Welch test with Games-Howell correction was used (*P* < 0.05). Significant differences prompted separate association analyses.

Prolonged recovery time of physical functioning was determined using a data-driven approach, dichotomizing recovery time based on exceeding the median and 75^th^ percentile, stratified by the type of surgical approach (not delayed/delayed).

Missing data were handled using stochastic multiple imputation with fully conditional specifications, with five imputed datasets generated. Only variables considered missing at random were imputed; variables missing not at random were not.

Multivariate logistic regression analyses were employed to assess associations between preoperative physical fitness and short-term postoperative outcomes. Results were presented as odds ratios (OR) with 95% confidence intervals (CI). All statistical tests were two-tailed, and a *P*-value <0.05 was considered statistically significant.

## Results

### Study Population Characteristics

**Figure 1.**
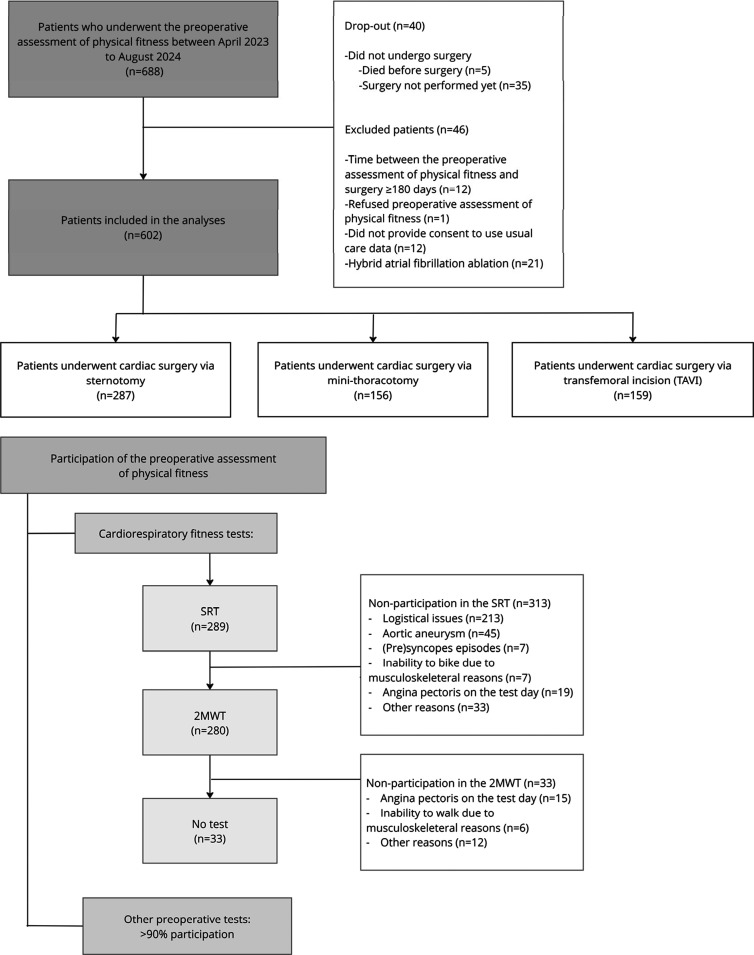
Flow chart study population

Data were retrospectively extracted from 688 patients, of whom 602 were included in the study. Forty patients were excluded due to non-receipt of surgery, and 46 patients were excluded for other reasons (Figure 1).

Of the 602 included patients, 289 performed the SRT to assess cardiorespiratory fitness, while 280 completed the 2MWT. Reasons for non-participation in the cardiorespiratory fitness tests are provided in Figure 1. The majority of surgeries were performed via sternotomy (47.7%), followed by transfemoral incision (TAVI) (26.4%) and mini-thoracotomy (25.9%). Detailed demographic and preoperative clinical characteristics, along with preoperative physical fitness outcomes, are summarized in Table 2, with an extended version including all pairwise comparisons available in Supplementary File 1.

**Table 2.** Preoperative Patient Characteristics

Variable	Total cohort (n = 602)		Sternotomy (n = 287)		Mini-thoracotomy (n = 156)		Transfemoral incision (TAVI) (n = 159)		*P*
Demographic characteristics									
Age (years)	70.0	(62.0–76.3)	67.0	(61.0–71.0)	64.5	(58.0–71.0)	78.0	(74.0–82.0)	* Ɨ
Sex									* Ɨ
Male	420	(69.8%)	221	(77.0%)	111	(71.2%)	88	(55.3%)	
Female	182	(30.2%)	66	(23.0%)	45	(28.8%)	71	(44.7%)	
Health-related characteristics									
BMI (kg/m^2^)	26.6	(24.2–29.2)	26.4	(24.2–29.8)	25.7	(23.7–30.0)	27.7	(24.7–30.0)	#
Diabetes mellitus	128	(21.3%)	56	(19.5%)	23	(14.7%)	48	(30.2%)	* Ɨ
COPD	47	(7.8%)	24	(9.1%)	9	(6.1%)	15	(9.4%)	
Smoking status									
Never smoked	232	(38.7%)	98	(57.2%)	65	(41.7%)	68	(42,8%)	
Current smoker	75	(12.5%)	37	(12.9%)	28	(17.9%)	10	(6.3%)	Ɨ
Former smoker	291	(48.5%)	149	(52.1%)	61	(39.4%)	81	(50.9%)	* Ɨ
LVEF (<50%)	107	(17.8%)	51	(17.8%)	30	(19.2%)	26	(16.4%)	
eGFR (mL/min/1.73 m^2^)	73.7	(60.2–88.2)	79.3	(64.2–90.0)	79.9	(67.4–90.0)	63.0	(49.3–73.5)	
EuroSCORE II	1.42	(0.91–2.60)	1.33	(0.87–2.51)	1.00	(0.68–1.46)	2.12	(1.48–3.18)	* # Ɨ
Surgery characteristics									* # Ɨ
Revascularization surgery	185	(30.7%)	124	(43.2%)	61	(39.1%)	/	/	
Valve(s) surgery	290	(48.2%)	54	(18.8%)	77	(49.4%)	159	(100%)	
Aortic aneurysm repair	44	(7.3%)	44	(15.3%)	/	/	/	/	
Combined surgery	75	(12.5%)	61	(21.3%)	14	(9.0%)	/	/	
Other	8	(1.3%)	4	(1.4%)	4	(2.6%)			
Preoperative physical fitness									
Cardiorespiratory fitness									
SRT WR_peak_^a^(W/kg)	2.4	(±0.9)	2.6	(±0.8)	2.8	(±0.9)	1.8	(±0.7)	* Ɨ
2MWT distance^b^(m)	173	(145–194)	179	(151–200)	173	(150–197)	158	(117–175)	* Ɨ
FitMáx VO_2peak_ (mL/kg/min)	18.3	(15.2–22.6)	19.2	(16.4–23.5)	20.2	(16.8–26.6)	14.8	(12.8–18.1)	* Ɨ
Muscle strength									
HGS (kPa)	79.1	(±20.8)	82.1	(±18.8)	83.7	(±21.8)	68.2	(±18.8)	* Ɨ
MIP (cm H_2_O)	76.1	(±27.1)	81.2	(±25.6)	78.6	(±26.7)	64.0	(±26.7)	* Ɨ
Functional mobility									
TUG (s)	6.5	(5.7–8.0)	6.2	(5.5–7.3)	6.2	(5.4–7.4)	8.1	(6.7–10.2)	* Ɨ
Frailty									
EFS (frail >6 points)	87	(14.8%)	26	(9.3%)	24	(15.8%)	37	(24.2%)	*
Health-related quality of live									
SF36 physical	44.2	(38.2–50.7)	45.7	(38.8–51.3)	45.1	(39.3–52.6)	41.2	(35.3–47.0)	* Ɨ
SF36 mental	46.7	(39.8–52.1)	47.3	(39.7–52.1)	45.6	(38.9–52.0)	46.4	(41.7–53.3)	

Data displayed as mean (±standard deviation), median (1st quartile-3rd quartile), or number (percentage). *P*-values were obtained using one-way ANOVA. or Kruskal–Wallis, or Chi-square test with post-hoc analyses. Significant difference (*P*-value < 0.05) between groups: * significant difference between sternotomy and transfemoral incision; # significant difference between sternotomy and mini-thoracotomy; Ɨ significant difference between mini-thoracotomy and transfemoral incision.

Abbreviations: ANOVA, analysis of variance; COPD, chronic obstructive pulmonary disease; EFS, Edmonton frailty scale; eGFR, estimated glomerular filtration rate; HGS, handgrip strength; LVEF, left ventricular ejection fraction; MIP, maximal inspiratory pressure; SF36, 36-item short form health survey; SRT, steep ramp test; TAVI, transcatheter aortic valve implantation; TUG, timed up-and-go; WR_peak_, work rate at peak exercise; QoL, quality of life; 2MWT, two-minute walk test.

^a^different sample size for the SRT: sternotomy n = 113; mini-thoracotomy n = 90; transfemoral incision n = 86.

^b^different sample size for the 2MWT: sternotomy n = 156; mini-thoracotomy n = 61; transfemoral incision n = 63.

Before performing logistic multivariate regression analysis, missing values for pre- and postoperative variables were imputed. Missing values for the SRT and the 2MWT were considered as missing data not at random which was attributed to contraindications and logistical reasons. Therefore, these two variables were not imputed. All other variables were complete following imputation. An overview of missing data is presented in the Supplementary File 2.

### Postoperative Outcomes

The median time to recovery of physical functioning was 3 days for patients undergoing sternotomy and mini-thoracotomy, while this was 1 day for those who underwent a transfemoral incision (*P* < 0.001) (Table 3).

**Table 3.** Postoperative Outcome Parameters

	Total cohort		Sternotomy		Mini-thoracotomy		Transfemoral incision (TAVI)		*P*
Recovery of physical functioning									
Time to mILAS = 0 (days)	3.0	(2.0–4.0)	3.0	(3.0–5.0)	3.0	(2.0–4.0)	1.0	(1.0–1.0)	* Ɨ
Delayed recovery of physical functioning									
Delayed (median cutoff)	220	(38.3%)	138	(52.5%)	59	(38.6%)	23	(14.7%)	* # Ɨ
Delayed (75^th^ percentile cutoff)	119	(20.8%)	68	(25.8%)	27	(17.6%)	23	(14.7%)	*
Postoperative in-hospital complications									
Occurrence of ≥1 complication	99	(16.4%)	71	(24.7%)	15	(9.8%)	13	(8.2%)	* #
Postoperative atrial fibrillation									
Occurrence of POAF	90	(20.2%)	75	(26.1%)	36	(23.5%)	15	(9.4%)	* Ɨ
Time between preoperative assessment of physical fitness and surgery									
Time (days)	51.0	(28.0–77.0)	48.0	(25.0–77.0)	65.5	(35.3–90.8)	44	(27.0–62.0)	# Ɨ

Data displayed as median (1st quartile-3rd quartile), or number (percentage). *P*-values were obtained using one-way ANOVA, or Kruskal-Wallis, or Chi-square test with post-hoc analyses. Significant difference (*P*-value < 0.05) between groups: * significant difference between sternotomy and transfemoral incision; # significant difference between sternotomy and mini-thoracotomy; Ɨ significant difference between mini-thoracotomy and transfemoral incision.

Abbreviations: mILAS, modified Iowa level of assistance scale; POAF, postoperative atrial fibrillation; TAVI, transcatheter aortic valve implantation.

Prolonged recovery, defined by the median recovery time cutoff, was observed in 52.5%, 38.6%, and 14.7% of patients undergoing sternotomy, mini-thoracotomy, and transfemoral incision, respectively. Using the 75th percentile cutoff, delayed recovery occurred in 25.8%, 17.6%, and 14.7% of these patients.

Postoperative in-hospital complications were reported in 24.7%, 9.8%, and 8.2% of patients undergoing sternotomy, mini-thoracotomy, and transfemoral incision, respectively. POAF was observed in 26.1%, 23.5%, and 9.4% of cases. A detailed description of postoperative outcomes is provided in Table 3 with an extended version including all pairwise comparisons available in Supplementary File 3. An overview of preoperative physical fitness stratified by postoperative outcome measures across different cardiac surgery procedures, are presented in Supplementary file 4-7.

### Logistic Multivariate Regression Analysis

Due to significant differences between the different surgical procedures, associations were analyzed separately for each surgical approach. Table 4 presents the results of the multivariate regression analysis, adjusted for potential confounders.

**Table 4.** Binary Multivariable Regression Analysis for Delayed Recovery of Physical Functioning, Postoperative In-Hospital Complications, and POAF in Patients Receiving Cardiac Surgery via (A) Sternotomy, (B) Mini-Thoracotomy, and (C) Transfemoral Incision (TAVI)

A) Via sternotomy (n = 287)												
	Delayed recovery of physical functioning						Postoperative in-hospital complications					
	Time to mILAS 0 ≥4 days (median)			Time to mILAS 0 ≥6 days (75^th^ percentile)			Occurrence of ≥1 complication			Postoperative AF		
	OR	95% CI	*P*	OR	95% CI	*P*	OR	95% CI	P	OR	95% CI	*P*
Cardiorespiratory fitness												
SRT WR_peak_ (W/kg)^a^	0.600	(0.308–1.170)	0.134	0.622	(0.278–1.392)	0.248	0.809	(0.372–1.757)	0.592	0.89	(0.443–1.784)	0.741
2MWT distance (m)^b^	0.992	(0.983–1.002)	0.110	0.990	(0.980–1.000)	0.051	0.992	(0.982–1.001)	0.084	1.001	(0.991–1.012)	0.778
FitMáx VO_2peak_ (mL/kg/min)	0.903	(0.856–0.953)	<0.001*	0.895	(0.827–0.969)	0.007*	0.929	(0.872–0.990)	0.024*	0.958	(0.903–1.017)	0.163
Muscle strength												
HGS (kPa)	0.990	(0.975–1.006)	0.217	0.996	(0.979–1.014)	0.687	0.993	(0.977–1.010)	0.445	1.001	(0.985–1.018)	0.874
MIP (cmH_2_O)	0.996	(0.996–1.004)	0.433	0.991	(0.978–1.004)	0.171	0.993	(0.981–1.006)	0.283	1.002	(0.990–1.014)	0.705
Functional mobility												
TUG (s)	1.213	(1.027–1.433)	0.023*	1.244	(1.048–1.478)	0.013*	1.194	(1.010–1.413)	0.038*	1.050	(0.891–1.238)	0.560
Frailty												
EFS (frail >6 points)	3.541	(1.355–9.251)	0.010*	3.306	(1.394–7.843)	0.007*	2.874	(1.222–6.763)	0.016*	1.282	(0.527–3.117)	0.584
Health-related quality of life												
SF36 physical	0.958	(0.929–0.988)	0.007*	0.956	(0.920–0.993)	0.021*	0.952	(0.918–0.987)	0.007*	0.994	(0.961–1.028)	0.712
SF36 mental	0.993	(0.967–1.020)	0.615	0.985	(0.955–1.016)	0.344	0.977	(0.947–1.008)	0.143	0.980	(0.951–1.010)	0.188

B) Via mini-thoracotomy (n = 156)												
	Delayed recovery of physical functioning						Postoperative in-hospital complications					
	Time to mILAS 0 ≥4 days (median)			Time to mILAS 0 ≥5 days (75^th^ percentile)			Occurrence of ≥1 complication			Postoperative AF		
	OR	95% CI	*P*	OR	95% CI	*P*	OR	95% CI	*P*	OR	95% CI	*P*
Cardiorespiratory fitness												
SRT WR_peak_ (W/kg)^a^	0.726	(0.360–1.464)	0.370	0.674	(0.298–1.524)	0.343	0.542	(0.146–2.017)	0.360	0.993	(0.967–1.020)	0.615
2MWT distance (m)^b^	0.994	(0.973–1.016)	0.592	0.992	(0.969–1.016)	0.533	0.960	(0.930–0.991)	0.012*	0.987	(0.966–1.008)	0.230
FitMáx VO_2peak_ (mL/kg/min)	0.993	(0.993–1.062)	0.828	0.952	(0.869–1.043)	0.293	0.909	(0.810–1.022)	0.110	1.020	(0.943–1.104)	0.621
Muscle strength												
HGS (kPa)	1.000	(0.981–1.018)	0.959	1.013	(0.990–1.036)	0.260	0.985	(0.956–1.014)	0.299	1.000	(0.980–1.020)	0.968
MIP (cm H_2_O)	0.992	(0.978–1.008)	0.332	0.987	(0.969–1.006)	0.182	0.991	(0.968–1.014)	0.417	1.003	(0.986–1.021)	0.695
Functional capacity												
TUG (s)	1.048	(0.847–1.297)	0.666	1.176	(0.913–1.514)	0.208	1.404	(1.035–1.903)	0.029*	1.008	(0.793–1.281)	0.949
Frailty												
EFS (frail >6 points)	1.001	(0.391–2.562)	0.999	1.266	(0.456–4.086)	0.578	1.253	(0.292–5.379)	0.762	0.952	(0.325–2.784)	0.928
Health-related quality of life												
SF36 physical	1.008	(0.964–1.054)	0.733	1.005	(0.950–1.062)	0.870	0.957	(0.894–1.025)	0.214	1.010	(0.960–1.064)	0.696
SF36 mental	0.989	(0.951–1.030)	0.594	0.999	(0.975–1.047)	0.953	1.011	(0.948–1.079)	0.731	1.015	(0.972–1.061)	0.499

C) Via transfemoral incision (TAVI) (n = 159)												
	Delayed recovery of physical functioning						Postoperative in-hospital complications					
	Time to mILAS 0 ≥2 days (median = 75^th^ percentile)						Occurrence of ≥1 complication			Postoperative AF		
	OR	95% CI	*P*				OR	95% CI	*P*	OR	95% CI	*P*
Cardiorespiratory fitness												
SRT WR_peak_ (W/kg)^a^	0.946	(0.332–2.822)	0.909				0.968	(0.332–2.822)	0.952	0.423	(0.111–1.602)	0.205
2MWT distance (m)^b^	1.005	(0.964–1.036)	0.654				0.999	(0.964–1.036)	0.960	1.006	(0.978–1.034)	0.691
FitMáx VO_2peak_ (mL/kg/min)	0.945	(0.862–1.185)	0.445				1.011	(0.862–1.185)	0.894	0.923	(0.768–1.110)	0.395
Muscle strength												
HGS (kPa)	0.945	(0.817–1.093)	0.445				1.014	(0.979–1.050)	0.442	0.971	(0.938–1.006)	0.104
MIP (cm H_2_o)	1.001	(0.974–1.028)	0.957				1.010	(0.983–1.036)	0.476	0.996	(0.969–1.023)	0.757
Functional capacity												
TUG (s)	0.957	(0.821–1.114)	0.569				0.834	(0.643–1.081)	0.170	0.995	(0.833–1.188)	0.958
Frailty												
EFS (frail >6 points)	0.681	(0.215–2.153)	0.513				0.640	0.156–2.617)	0.534	1.260	(0.351–4.517)	0.723
Health-related quality of life												
SF36 physical	0.995	(0.933–1.061)	0.874				1.015	(0.944–1.092)	0.685	1.033	(0.953–1.120)	0.423
SF36 mental	1.014	(0.961–1.069)	0.619				1.057	(0.975–1.146)	0.181	1.003	(0.937–1.073)	0.936

Multivariate logistic regression analysis, variable corrected for the potential confounders: Euroscore II, body mass index, age, and sex.

*Indicates a statistically significant *P*-value (*P* < 0.05).

Abbreviations: EFS, Edmonton frailty scale; HGS, handgrip strength; LVEF, left ventricular ejection fraction; MIP, maximal inspiratory pressure; n, sample size; OR, odds ratio; SF36, 36-item short form health survey; SRT, steep ramp test; TAVI, transcatheter aortic valve implantation; TUG, timed up-and-go; WR_peak_, work rate at peak exercise; 2MWT, two-minute walk test.

^a^different sample size for the SRT: sternotomy n=113; mini-thoracotomy n= 90; transfemoral incision n=86.

^b^different sample size for the 2MWT: sternotomy n=156; mini-thoracotomy n=61; transfemoral incision n=63.

#### Sternotomy

Multivariate logistic regression showed significant associations between preoperative patient-reported cardiorespiratory fitness (FitMáx) and delayed recovery (median cutoff: OR = 0.903, 95% CI: 0.856–0.953, *P*=<0.001; 75^th^ percentile cutoff: OR = 0.895, 95% CI: 0.827–0.969, *P* = 0.007) and postoperative in-hospital complications (OR = 0.929, 95% CI: 0.872–0.990, *P* = 0.024). Each 1 mL/kg/min increase in estimated VO_2peak_ reduced the odds of delayed recovery (median cutoff: 9.7%, 75^th^ percentile cutoff: 10.5%) and complications (7.1%).

Preoperative functional mobility (TUG) was significantly associated with delayed recovery (median cutoff: OR = 1.213, 95% CI: 1.027–1.433, *P* = 0.023; 75th percentile cutoff: OR = 1.244, 95% CI: 1.048–1.478, *P* = 0.013) and complications (OR = 1.194, 95% CI: 1.010–1.413, *P* = 0.038). Each additional second on the TUG increased the odds of these outcomes by 19–24%.

Preoperative frailty (EFS >6) was linked to a threefold increased risk of delayed recovery (median cutoff: OR = 3.541, 95% CI: 1.355–9.251; *P* = 0.010; 75^th^ percentile cutoff: OR = 3.306, 95% CI: 1.394–7.843, *P* = 0.007) and complications (OR = 2.874, 95% CI: 1.222–6.763, *P* = 0.016).

Better preoperative physical health-related QoL (SF-36) was associated with lower odds of delayed recovery (median cutoff: OR = 0.958, 95% CI: 0.929–0.988, *P* = 0.007; 75th percentile cutoff: OR = 0.956, 95% CI: 0.920–0.993, *P* = 0.021) and complications (OR = 0.952, 95% CI: 0.918–0.987, *P* = 0.007), with a 10-point increase reducing risk by 34.9%, 36.2%, and 38.9%, respectively.

No associations were found between preoperative SRT, 2MWT, HGS, MIP, SF-36 mental health, and postoperative delayed recovery or complications. No preoperative physical fitness parameter was associated with POAF.

#### Mini-Thoracotomy

Multivariate regression analysis identified significant associations between preoperative 2MWT and TUG performance with postoperative in-hospital complications. Each additional 10 meters walked in the 2MWT was associated with a 33.4% reduction in the odds of complications (OR = 0.960, 95% CI: 0.930–0.991, *P* = 0.012), while each additional second required to complete the TUG was linked to a 40.4% increase in risk (OR = 1.404, 95% CI 1.035–1.903, *P* = 0.029). No other preoperative physical fitness parameter were significantly associated with postoperative in-hospital complications. No preoperative physical fitness parameter was associated with delayed recovery and POAF.

#### Transfemoral Incision (TAVI)

No significant associations were found between preoperative physical fitness and postoperative outcomes.

## Discussion

This study investigated the association between preoperative physical fitness and short-term postoperative outcomes in patients undergoing elective cardiac surgery. Higher patient-reported preoperative cardiorespiratory fitness, functional mobility, physical health-related QoL, and absence of frailty were significantly associated with faster recovery of physical functioning and fewer in-hospital complications in patients undergoing cardiac surgery via sternotomy. For patients undergoing mini-thoracotomy, significant associations were found between preoperative cardiorespiratory fitness and functional mobility, and in-hospital complications. No associations were found for patients undergoing transfemoral incision (TAVI).

### Key Findings by Surgical Approach

#### Sternotomy

A key finding is the significant relationship between preoperative cardiorespiratory fitness, estimated by the FitMáx questionnaire, and postoperative outcomes. Patients with lower estimated VO_2peak_ exhibited a higher risk of delayed recovery and in-hospital complications, aligning with previous research.^3^ These results suggest the FitMáx questionnaire could serve as a preoperative screening tool for identifying high-risk patients.^17^

Although objective fitness measures like the 2MWT and SRT did not reach statistical significance, trends suggest clinical relevance. For instance, a 10-meter increase in the 2MWT was associated with an 8% reduction in delayed recovery, and a 0.1 W/kg increase in SRT WR_peak_ was linked to a 5% reduction in delayed recovery, consistent with prior studies.^4,28^

The finding that the FitMáx questionnaire outperformed objective measures might seem as unexpected and possibly caused by the wider data availability. However, even when analyzed by group, the FitMáx questionnaire continued to outperform the objective measures (Supplementary file 8). Future research should explore the relationship between objective measures and postoperative outcomes, using a larger sample size.

The TUG was associated with delayed recovery in sternotomy patients. Prior research suggests that cardiac patients can improve TUG times by 0.8 seconds with prehabilitation, while those without intervention improve by only 0.1 seconds.^29^ An additional 0.8 seconds on the TUG test was associated with an increase in the odds of delayed recovery and complications by 15–19%. Additionally, frailty, measured by the EFS, was significantly linked to poor postoperative outcomes, highlighting the importance of frailty screening in preoperative assessments.^30^

#### Mini-Thoracotomy

Limited data exist on preoperative physical fitness and postoperative outcomes in mini-thoracotomy patients. This study found significant associations between preoperative cardiorespiratory fitness and functional mobility and the risk of postoperative complications, indicating the relevance of physical fitness even in less invasive procedures. Larger studies are required to confirm these findings.

#### Transfemoral Incision (TAVI)

No significant associations were observed between preoperative fitness and short-term outcomes in transfemoral incision patients. The low incidence of complications in this group suggests that the minimally invasive nature of the procedure may lessen the impact of preoperative physical fitness. However, previous research has linked frailty with long-term outcomes, such as 1-year mortality.^31^

#### Methodological Considerations

Delayed recovery of physical functioning was defined using data-driven cut-offs based on the median and the 75th percentile, as no standard cut-off value exists. The consistency of associations across both cut-offs strengthens the robustness and predictive relevance of the preoperative measures.

### Clinical Implications

This study provides strong evidence for integrating comprehensive physical fitness assessments into routine preoperative care for cardiac surgery, particularly for more invasive procedures. By incorporating preoperative physical fitness variables, we can identify high-risk patients who may benefit most from targeted and personalized interventions to optimize their fitness and improve postoperative outcomes.

Current literature on prehabilitation in cardiac patients has reported inconsistent effects, largely due to the use of generalized programs applied to all patients undergoing cardiac surgery, without adequately considering individual risk factors. Previous studies did not stratify by fitness, and trained all patients^32^ or only applied broad criteria like age^29^ or frailty.^33^

Our findings directly address the gap by establishing a clear association between specific modifiable risk factors and postoperative complication and delayed recovery across different types and invasiveness of cardiac surgery. This evidence strongly supports a personalized approach rooted in these specific, modifiable risk factors. The use of a comprehensive fitness assessment allows for tailored interventions. For example, individuals identified with low VO_2_peak (FitMáx), slow TUG times, or high EFS scores are at a higher risk for adverse short-term outcomes and should be prioritized for structured, supervised prehabilitation programs aimed at improving cardiorespiratory fitness and functional capacity. In contrast, low-risk patients may not need intensive prehabilitation but could benefit from educational interventions (e.g., guidance on safe activity levels) to maintain their status, allowing for more efficient resource allocation towards higher-risk patients.

The FitMáx questionnaire and EFS offer practical, cost-effective tools for initial risk assessment, while the 2MWT and TUG could serve as valuable and feasible assessments, particularly for mini-thoracotomy patients. The stronger association between physical fitness and postoperative outcomes observed in more invasive procedures (e.g., sternotomy) highlights the need for further research, especially focusing on this cohort.

Ultimately, incorporating these physical fitness variables into current STS/EuroScore risk models could enhance their predictive power, helping to identify high-risk patients undergoing sternotomy. Developing a validated prediction model based on these modifiable risks would be the next step to facilitate shared decision-making and enable the optimization of individual patient status prior to surgery, ultimately leading to improved recovery trajectories.

#### Strengths and Limitations

A major strength of this study is the comprehensive assessment of physical fitness, utilizing validated, practical tests that enabled the exploration of various fitness domains and postoperative outcomes. Furthermore, the diverse surgical population enhances generalizability. Conversely, the necessity of stratifying the analysis by surgical approach reduced the subgroup sizes for mini-thoracotomy and TAVI, which limits the statistical power in these groups.

#### Conclusion

Better preoperative physical fitness, including cardiorespiratory fitness, functional mobility, frailty, and physical health-related quality of life, was associated with a reduced risk of adverse postoperative outcomes in patients undergoing cardiac surgery via a sternotomy approach. For patients undergoing mini-thoracotomy, some associations were found that require larger studies for confirmation, while no associations were found for patients undergoing a transfemoral incision (TAVI).

## Supplemental Material

Supplemental Material - Preoperative Physical Fitness and Short-Term Postoperative Outcomes in Patients Undergoing Elective Cardiac Surgery: A Retrospective Cohort Study Supplemental Material for Preoperative Physical Fitness and Short-Term Postoperative Outcomes in Patients Undergoing Elective Cardiac Surgery: A Retrospective Cohort Study by Zoe van der Hoofd, Hanneke C. van Dijk-Huisman, Bart C. Bongers, Bart Scheenstra, Jos Maessen, and Antoine F. Lenssen in Seminars in Cardiothoracic and Vascular Anesthesia
