# Supplementary material for: Preoperative Physical Fitness and Short-Term Postoperative Outcomes in Patients Undergoing Elective Cardiac Surgery: A Retrospective Cohort Study
Source: Semin Cardiothorac Vasc Anesth. 2026 Feb 21;30(3):160–71. doi: 10.1177/10892532261427827 (PMC13457777; doi:10.1177/10892532261427827)
Supplement: Supplementary file 1 [file sj-pdf-1-scv-10.1177_10892532261427827.pdf]

Supplementary File 1 – Preoperative patient characteristics

Variable	Total cohort (n=602)		Sternotomy (n=287)		Mini-thoracotomy (n=156)		Transfemoral incision (TAVI) (n=159)		P	P-value - Difference between sternotomy and transfemoral incision	P-value - Difference between sternotomy and mini-thoracotomy	P-value - Difference between mini-thoracotomy and transfemoral incision
**Demographic characteristics**												
**Age (years)**	70.0	(62.0-76.3)	67.0	(61.0-71.0)	64.5	(58.0-71.0)	78.0	(74.0-82.0)	* ɫ	<0.001	0.147	<0.001
**Sex**									* ɫ	<0.001	0.525	0.012
Male	420	(69.8%)	221	(77.0%)	111	(71.2%)	88	(55.3%)				
Female	182	(30.2%)	66	(23.0%)	45	(28.8%)	71	(44.7%)				
**Health-related characteristics**												
**BMI (kg/m^2)**	26.6	(24.2-29.2)	26.4	(24.2-29.8)	25.7	(23.7-30.0)	27.7	(24.7-30.0)	#	0.350	0.690	0.044
**Diabetes mellitus**	128	(21.3%)	56	(19.5%)	23	(14.7%)	48	(30.2%)	* ɫ	0.090	1.000	0.003
**COPD**	47	(7.8%)	24	(9.1%)	9	(6.1%)	15	(9.4%)		1.000	1.000	1.000
**Smoking status**												
Non smoker	232	(38.7%)	98	(57.2%)	65	(41.7%)	68	(42,8%)		0.468	0.768	1.000
Current smoker	75	(12.5%)	37	(12.9%)	28	(17.9%)	10	(6.3%)	ɫ	0.087	0.441	0.003
Former smoker	291	(48.5%)	149	(52.1%)	61	(39.4%)	81	(50.9%)	* ɫ	0.048	0.105	0.003
**LVEF (<50%)**	107	(17.8%)	51	(17.8%)	30	(19.2%)	26	(16.4%)		1.000	0.726	1.000
**eGFR (mL/min/1.73m^2)**	73.7	(60.2-88.2)	79.3	(64.2-90.0)	79.9	(67.4-90.0)	63.0	(49.3-73.5)		<0.001	0.830	<0.001

**EuroSCORE II**	1.42	(0.91-2.60)	1.33	(0.87-2.51)	1.00	(0.68-1.46)	2.12	(1.48-3.18)	* # ƚ	0.013	0.006	<0.001
**Surgery characteristics**									* # ƚ	<0.001	<0.001	<0.001
Revascularization surgery	185	(30.7%)	124	(43.2%)	61	(39.1%)	/	/				
Valve(s) surgery	290	(48.2%)	54	(18.8%)	77	(49.4%)	159	(100%)				
Aortic aneurysm repair	44	(7.3%)	44	(15.3%)	/	/	/	/				
Combined surgery	75	(12.5%)	61	(21.3%)	14	(9.0%)	/	/				
Other	8	(1.3%)	4	(1.4%)	4	(2.6%)						
**Preoperative physical fitness**												
Cardiorespiratory fitness												
**SRT WR$_{peak}$ ◊ (W/kg)**	2.4	(±0.9)	2.6	(±0.8)	2.8	(±0.9)	1.8	(±0.7)	* ƚ	<0.001	0.244	<0.001
**2MWT distance ◊◊ (m)**	173	(145-194)	179	(151-200)	173	(150-197)	158	(117-175)	* ƚ	<0.001	1.000	0.010
**FitMáx VO$_{2peak}$ (mL/kg/min)**	18.3	(15.2-22.6)	19.2	(16.4-23.5)	20.2	(16.8-26.6)	14.8	(12.8-18.1)	* ƚ	<0.001	0.621	<0.001
Muscle strength												
**HGS (kPa)**	79.1	(±20.8)	82.1	(±18.8)	83.7	(±21.8)	68.2	(±18.8)	* ƚ	<0.001	0.706	<0.001
**MIP (cm H_2O)**	76.1	(±27.1)	81.2	(±25.6)	78.6	(±26.7)	64.0	(±26.7)	* ƚ	<0.001	0.607	<0.001
Functional mobility												

**TUG (s)**	6.5	(5.7-8.0)	6.2	(5.5-7.3)	6.2	(5.4-7.4)	8.1	(6.7-10.2)	* ƚ	<0.001	1.000	<0.001
Frailty												
**EFS (frail >6 points)**	87	(14.8%)	26	(9.3%)	24	(15.8%)	37	(24.2%)	*	<0.001	0.126	0.201
Health-related quality of live												
**SF36 physical**	44.2	(38.2-50.7)	45.7	(38.8-51.3)	45.1	(39.3-52.6)	41.2	(35.3-47.0)	* ƚ	<0.001	1.000	<0.001
**SF36 mental**	46.7	(39.8-52.1)	47.3	(39.7-52.1)	45.6	(38.9-52.0)	46.4	(41.7-53.3)		1.000	1.000	1.000

*Data displayed as mean (±standard deviation), median (interquartile range), or number (percentage). P-values were obtained using one-way ANOVA. or Kruskal-Wallis, or Chi-square test with post-hoc analyses. Significant difference (p-value < 0.05) between groups: * significant difference between sternotomy and transfemoral incision; # significant difference between sternotomy and mini-thoracotomy; ƚ significant difference between mini-thoracotomy and transfemoral incision.*

Abbreviations: ANOVA, analysis of variance; COPD, chronic obstructive pulmonary disease; EFS, Edmonton frailty scale; eGFR, estimated glomerular filtration rate; HGS, handgrip strength; LVEF, left ventricular ejection fraction; MIP, maximal inspiratory pressure; SF36, 36-item short form health survey; SRT, steep ramp test; TAVI, transcatheter aortic valve implantation; TUG, timed up-and-go; WR_{peak}*, work rate at peak exercise; QoL, quality of life; 2MWT, two-minute walk test.*

◊ different sample size for the SRT: sternotomy n=113; mini-thoracotomy n= 90; transfemoral incision n=86.

◊◊ different sample size for the 2MWT: sternotomy n=156; mini-thoracotomy n=61; transfemoral incision n=63.

Supplementary file 2 – Missing data

Variable	Total cohort		Sternotomy		Mini-thoracotomy		Transfemoral incision	
	Complete (n)	Missing (n,%)	Complete (n)	Missing (n,%)	Complete (n)	Missing (n,%)	Complete (n)	Missing (n,%)
**SRT**	289	313 (52.0%)	113	174 (60.6%)	90	66 (42.3%)	86	73 (45.9%)
**SRT incl. AP**	300	302 (50.2%)	122	165 (57.5%)	91	65 (41.7%)	87	72 (45.3%)
**MWT**	280	322 (53.5%)	156	131 (45.6%)	61	95 (60.9%)	63	96 (60.4%)
**FitMáx**	586	16 (2.7%)	281	6 (2.1%)	151	5 (3.2%)	154	5 (3.1%)
**HGS**	601	1 (0.2%)	286	1 (0.3%)	156	0	159	0
**MIP**	561	41 (6.8%)	266	21 (7.3%)	150	6 (3.8%)	145	14 (8.8%)
**TUG**	592	10 (1.7%)	282	5 (1.7%)	156	0 (0%)	154	5 (3.1%)
**EFS**	586	16 (2.7%)	281	6 (2.1%)	152	4 (2.6%)	153	6 (3.8%)
**SF36 physical**	547	55 (9.1%)	267	20 (7.0%)	147	9 (5.8%)	133	26 (16.4%)
**SF36 mental**	547	55 (9.1%)	267	20 (7.0%)	147	9 (5.8%)	133	26 (16.4%)
**mILAS**	574	28 (4.7%)	265	22 (7.7%)	153	3 (1.9%)	156	3 (1.9%)

Data displayed as number (percentage)

Abbreviations: EFS, Edmonton frailty scale; HGS, handgrip strength; LVEF, left ventricular ejection fraction; MIP, maximal inspiratory pressure; n, sample size; SF36, medical outcome study 36-item short form; SRT, steep ramp test; TUG, timed up-and-go; WRpeak, work rate at peak exercise; QoL, quality of life; 2MWT, two-minute walk test.

Supplementary file 3 – Postoperative outcome parameters

	Total cohort		Sternotomy		Mini-thoracotomy		Transfemoral incision (TAVI)		P	P-value - Difference between sternotomy and transfemoral incision	P-value - Difference between sternotomy and mini-thoracotomy	P-value - Difference between mini-thoracotomy and transfemoral incision
**Recovery of physical functioning**												
Time to mILAS = 0 (days)	3.0	(2.0-4.0)	3.0	(3.0-5.0)	3.0	(2.0-4.0)	1.0	(1.0-1.0)	* ƚ	<0.001	0.106	<0.001
**Delayed recovery of physical functioning**												
Delayed (median cutoff)	220	(38.3%)	138	(52.5%)	59	(38.6%)	23	(14.7%)	* # ƚ	<0.001	0.024	<0.001
Delayed (75th percentile cutoff)	119	(20.8%)	68	(25.8%)	27	(17.6%)	23	(14.7%)	*	0.039	0.171	1.000
**Postoperative in-hospital complications**												
Occurrence of ≥1 complication	99	(16.4%)	71	(24.7%)	15	(9.8%)	13	(8.2%)	* #	<0.001	<0.001	1.000
**Postoperative atrial fibrillation**												
Occurrence of POAF	90	(20.2%)	75	(26.1%)	36	(23.5%)	15	(9.4%)	* ƚ	<0.001	1.000	<0.001
**Time between preoperative assessment of physical fitness and surgery**												
Time (days)	51.0	(28.0-77.0)	48.0	(25.0-77.0)	65.5	(35.3-90.8)	44	(27.0-62.0)	# ƚ	0.947	<0.001	<0.001

*Data displayed as median (1st quartile-3rd quartile), or number (percentage). P-values were obtained using one-way ANOVA, or Kruskal-Wallis, or Chi-square test with post-hoc analyses. Significant difference (p-value < 0.05) between groups: * significant difference between sternotomy and transfemoral incision; # significant difference between sternotomy and mini-thoracotomy; ƚ significant difference between mini-thoracotomy and transfemoral incision.*

Abbreviations: mILAS, modified Iowa level of assistance scale; POAF, postoperative atrial fibrillation; TAVI, transcatheter aortic valve implantation.

Supplementary file 4 - Comparison of pre-operative physical fitness parameters stratified by post-operative functional recovery (median cutoff) across different incision types

	**Sternotomy**					**Mini-thoracotomy**					**Transfemoral incision (TAVI)**				
	Normal		Delayed		P	Normal		Delayed		P	Normal		Delayed		P
	Time to mILAS 0 <3d		Time to mILAS 0 ≥4d			Time to mILAS 0 <3d		Time to mILAS 0 ≥4d			Time to mILAS 0 <1d		Time to mILAS 0 ≥2d		
Cardiorespiratory fitness															
**SRT◊ (WRpeak/kg)**	2.70	(±0.80)	2.40	(±0.72)	0.057	2.88	(±0.84)	2.65	(±0.67)	0.249	1.78	(±0.72)	1.72	(±0.80)	0.783
**2MWT◊◊ (m)**	180.0	(44)	175.5	(44)	0.172	175.0	(43)	172.5	(59)	0.266	160.0	(60)	145.0	(55)	0.374
**FitMáx (mL/kg/min)**	20.39	(9.88)	18.01	(1.30)	0.002	22.19	(9.87)	19.41	(10.07)	0.229	14.79	(5.89)	14.73	(4.70)	0.500
Muscle strength															
**HGS (kPa)**	84.80	(±20.12)	79.73	(±17.56)	0.030	85.81	(±21.47)	81.17	(±22.49)	0.203	68.74	(±18.38)	67.09	(±21.70)	0.698
**MIP (cmH_2O)**	84.08	(±26.15)	78.50	(±25.54)	0.093	82.37	(±26.74)	73.30	(±25.95)	0.047	64.98	(±27.32)	59.19	(±24.83)	0.366
Functional capacity															
**TUG (s)**	6.05	(1.57)	6.36	(1.95)	0.030	6.02	(2.00)	6.23	(1.97)	0.248	7.94	(3.43)	8.57	(2.53)	0.770
Frailty															
**EFS (frail >6 points)**	6	(4.8%)	19	(14.3 %)	0.009	13	(14.3%)	11	(18.6%)	0.477	32	(24.1%)	4	(18.2%)	0.489
Quality of live															
**SF36 physical**	46.15	(±7.98)	43.81	(±8.26)	0.025	45.23	(±7.61)	45.12	(±9.75)	0.944	41.50	(±7.74)	40.53	(±9.39)	0.633
**SF36 mental**	45.93	(±9.26)	46.22	(±9.45)	0.812	46.10	(±8.84)	45.28	(±9.45)	0.598	46.08	(±9.28)	45.27	(±7.39)	0.812

Data displayed as mean (± standard deviation), median (interquartile range), or number (percentage). P-values were obtained using independence t-test, or chi-square test.

Abbreviations: EFS, Edmonton frailty scale; HGS, handgrip strength; LVEF, left ventricular ejection fraction; MIP, maximal inspiratory pressure; n, sample size; SF36, medical outcome study 36-item short form; SRT, steep ramp test; TUG, timed up-and-go; WRpeak, work rate at peak exercise; QoL, quality of life; 2MWT, two-minute walk test.

◊ different sample size for the SRT: sternotomy n=113; mini-thoracotomy n= 90; transfemoral incision n=86

◊◊ different sample size for the 2MWT: sternotomy n=156; mini-thoracotomy n=61; transfemoral incision n=63.

Supplementary file 5 - Comparison of pre-operative physical fitness parameters stratified by post-operative functional recovery (75th percentile cutoff) across different incision types

	**Sternotomy**					**Mini-thoracotomy**					**Transfemoral incision (TAVI)**				
	Normal		Delayed			Normal		Delayed			Normal		Delayed		
	Time to mILAS 0 <5d		Time to mILAS 0 ≥6d		P	Time to mILAS 0 <4d		Time to mILAS 0 ≥5d		P	Time to mILAS 0 <1d		Time to mILAS 0 ≥2d		P
Cardiorespiratory fitness															
**SRT◊ (WRpeak/kg)**	2.62	(±0.81)	2.29	(±0.47)	0.022	2.86	(±0.86)	2.45	(±1.01)	0.094	1.78	(±0.72)	1.72	(±0.80)	0.783
**2MWT◊◊ (m)**	180.0	(±41)	174.5	(±70)	0.086	172.0	(±42)	174.0	(±60)	0.712	160.0	(±60)	145.0	(±55)	0.156
**FitMáx (mL/kg/min)**	19.75	(8.10)	17.56	(3.96)	<0.001	20.63	(10.13)	18.05	(9.35)	0.100	14.79	(5.89)	14.73	(4.70)	0.330
Muscle strength															
**HGS (kPa)**	83.19	(±19.38)	74.57	(±17.66)	0.085	83.55	(±20.79)	86.22	(±26.90)	0.567	68.74	(±18.38)	67.09	(±21.70)	0.565
**MIP (cmH_2O)**	82.97	(±25.83)	75.19	(±25.83)	0.041	80.29	(±27.38)	73.19	(±23.06)	0.220	64.98	(±27.32)	59.19	(±24.83)	0.366
Functional capacity															
**TUG (s)**	6.11	(1.70)	6.55	(2.16)	0.017	6.02	(1.91)	6.68	(1.87)	0.179	7.94	(3.43)	8.57	(2.53)	0.770
Frailty															
**EFS (frail >6 points)**	13	(6.7%)	12	(18.5%)	0.006	18	(14.6%)	6	(22.2%)	0.330	32	(25.2%)	4	(17.4%)	0.430
Quality of live															
**SF36 physical**	45.56	(±8.16)	43.04	(±7.99)	0.036	45.34	(±8.04)	44.55	(±10.30)	0.711	41.50	(±7.74)	40.53	(±9.39)	0.653
**SF36 mental**	46.13	(±8.83)	45.48	(±10.72)	0.634	45.79	(±8.92)	45.76	(±9.82)	0.990	46.08	(±9.28)	45.27	(±7.39)	0.891

Data displayed as mean (±standard deviation), median (interquartile range), or number (percentage). P-values were obtained using independence t-test, or chi-square test.

Abbreviations: EFS, Edmonton frailty scale; HGS, handgrip strength; LVEF, left ventricular ejection fraction; MIP, maximal inspiratory pressure; n, sample size; SF36, medical outcome study 36-item short form; SRT, steep ramp test; TUG, timed up-and-go; WRpeak, work rate at peak exercise; QoL, quality of life; 2MWT, two-minute walk test.

◊ different sample size for the SRT: sternotomy n=113; mini-thoracotomy n= 90; transfemoral incision n=86

◊◊ different sample size for the 2MWT: sternotomy n=156; mini-thoracotomy n=61; transfemoral incision n=63.

Supplementary file 6 - Comparison of pre-operative physical fitness parameters stratified by the occurrence post-operative in-hospital complications across different incision types

	**Sternotomy**					**Mini-thoracotomy**					**Transfemoral incision (TAVI)**				
	Postoperative complications				P	Postoperative complications				P	Postoperative complications				P
	<1 complication		≥1 complication			<1 complication		≥1 complication			<1 complication		≥1 complication		
Cardiorespiratory fitness															
**SRT (WRpeak/kg)**	2.61	(±0.78)	2.47	(±0.74)	0.463	2.80	(±0.93)	2.51	(±0.26)	0.056	1.75	(±0.74)	1.89	(±0.61)	0.537
**2MWT (m)**	180.0	(±45)	176.0	(±72)	0.322	178.5	(±42)	130.0	(±47)	0.005	160.0	(±59)	135.0	(±51)	0.317
**FitMáx (mL/kg/min)**	19.68	(8.26)	18.39	(4.44)	0.059	20.37	(10.41)	18.62	(5.52)	0.335	14.79	(5.90)	15.17	(2.81)	0.822
Muscle strength															
**HGS (kPa)**	82.72	(±18.22)	80.04	(±20.30)	0.298	84.02	(±22.25)	80.87	(±19.69)	0.599	68.23	(±18.74)	67.93	(±19.90)	0.954
**MIP (cmH_2O)**	82.27	(±25.19)	77.73	(±26.94)	0.221	79.14	(±27.27)	73.71	(±21.91)	0.473	64.10	(±27.33)	63.43	(±21.07)	0.929
Functional capacity															
**TUG (s)**	6.16	(1.53)	6.47	(7.87)	0.134	6.11	(1.97)	6.68	(2.96)	0.093	8.08	(3.64)	8.43	(2.53)	0.881
Preoperative frailty															
**EFS (frail >6 points)**	15	(7.1%)	11	(15.9%)	0.027	21	(15.6%)	3	(21.4%)	0.569	34	(24.6%)	3	(20.0%)	0.690
Quality of live															
**SF36 physical**	45.59	(±8.03)	42.94	(±11.11)	0.021	45.43	(±8.26)	42.22	(±9.63)	0.176	41.29	(±7.96)	40.62	(±7.96)	0.799
**SF36 mental**	46.46	(±8.97)	44.92	(±9.89)	0.238	45.68	(±9.13)	45.73	(±8.58)	0.984	45.96	(±9.11)	46.20	(±6.63)	0.935

Data displayed as mean (±standard deviation), median (interquartile range), or number (percentage). P-values were obtained using independence t-test, or chi-square test.

Abbreviations: n, sample size; SD, standard deviation; IQR, interquartile range; kg, kilogram; SRT, Steep Ramp Test; WRpeak, work rate at peak exercise; SRT incl. AP; Steep ramp test inclusive data of patients who did not performed the test because of angina pectoris on the screenings day; 2MWT, two-minute walk test; m, meter; HGS, hand grip strength; kPa, kilopascal; MIP, maximum inspiratory pressure; cmH2O, unit centimeter of water; TUG, timed up and go; EFS, Edmonton frailty scale; SF36, Medical Outcome Study 36-Item Short Form.

◊ different sample size for the SRT: sternotomy n=113; mini-thoracotomy n= 90; transfemoral incision n=86

◊◊ different sample size for the 2MWT: sternotomy n=156; mini-thoracotomy n=61; transfemoral incision n=63.

Supplementary file 7- Comparison of pre-operative physical fitness parameters stratified by the occurrence of POAF across different incision types

	**Sternotomy**					**Mini-thoracotomy**					**Transfemoral incision (TAVI)**				
	POAF				P	POAD				P	POAF				P
	no		yes			no		yes			no		yes		
Cardiorespiratory fitness															
**SRT (WRpeak/kg)**	2.59	(±0.80)	2.58	(±0.72)	0.937	2.78	(±0.91)	2.79	(±0.89)	0.937	1.79	(±0.73)	1.48	(±0.61)	0.280
**2MWT (m)**	180	(53)	176.5	(34)	0.750	183.0	(43)	161.5	(42)	0.076	160.0	(58)	150.0	(45)	0.708
**FitMáx (mL/kg/min)**	19.45	(7.68)	18.64	(5.61)		20.66	(10.07)	19.45	(8.31)		14.73	(5.62)	15.97	(3.92)	
Muscle strength															
**HGS (kPa)**	82.52	(±19.71)	80.76	(±15.80)	0.487	83.65	(±21.00)	83.92	(±25.02)	0.949	69.19	(±18.61)	58.67	(±18.45)	0.038
**MIP (cmH_2O)**	81.45	(±25.41)	80.49	(±26.44)	0.789	78.11	(±27.64)	80.42	(±23.94)	0.331	64.67	(±26.35)	57.62	(±30.76)	0.366
Functional capacity															
**TUG (s)**	6.12	(1.68)	6.71	(1.49)	0.422	6.02	(2.07)	6.27	(1.91)	0.527	8.00	(3.44)	8.66	(2.90)	0.895
Preoperative frailty															
**EFS (frail >6 points)**	8	(11.0%)	18	(8.7%)	0.559	6	(16.7%)	18	(15.4%)	0.917	4	(26.7%)	33	(23.9%)	0.813
Quality of live															
**SF36 physical**	45.04	(±8.30)	44.66	(±7.46)	0.738	45.32	(±8.32)	44.45	(±8.1)	0.602	40.92	(±7.99)	44.14	(±7.0)	0.166
**SF36 mental**	46.48	(±8.88)	44.96	(±10.05)	0.230	45.61	(±9.00)	45.92	(±9.33)	0.862	46.00	(±8.68)	45.79	(±11.39)	0.938

Data displayed as mean (±standard deviation), median (interquartile range), or number (percentage). P-values were obtained using independence t-test, or chi-square test.

Abbreviations: n, sample size; SD, standard deviation; IQR, interquartile range; kg, kilogram; SRT, Steep Ramp Test; WRpeak, work rate at peak exercise; SRT incl. AP; Steep ramp test inclusive data of patients who did not performed the test because of angina pectoris on the screenings day; 2MWT, two-minute walk test; m, meter; HGS, hand grip strength; kPa, kilopascal; MIP, maximum inspiratory pressure; cmH2O, unit centimeter of water; TUG, timed up and go; EFS, Edmonton frailty scale; SF36, Medical Outcome Study 36-Item Short Form.

◊ different sample size for the SRT: sternotomy n=113; mini-thoracotomy n= 90; transfemoral incision n=86

◊◊ different sample size for the 2MWT: sternotomy n=156; mini-thoracotomy n=61; transfemoral incision n=63.

Supplementary file 8 – Subanalysis of patients undergoing cardiac surgery via sternotomy and the comparison of different cardiorespiratory fitness measures

A) Comparison of characteristics between the different groups of cardiorespiratory fitness measures (SRT versus 2MWT versus no test)

	SRT-group (n=113)		*2MWT-group* (n=156)		*No test-group* (n=18)		P-value
**Age** (years)	67.0	(12)	67.0	(14)	66.5	(12)	0.917
**EuroScoreII**	1.27	(1.19)	1.34	(2.12)	1.44	(1.87)	0.020*
**BMI** (kg/m^2)	26.67	(5.58)	26.30	(5.68)	27.19	(5.94)	0.821
**FitMáx VO_{2peak}** (ml/kg/min)	18.72	(7.55)	19.73	(6.77)	16.11	(7.85)	0.085
**EFS** (frail > 6 points)	10	(8.8%)	15	(9.6%)	1	(5.6%)	0.901
**TUG** (s)	6.31	(1.81)	6.11	(1.77)	6.55	(3.11)	0.489
**HGS** (kPa)	81.87	(±17.86)	82.37	(±19.16)	80.56	(±21.61)	0.919
**MIP** (cmH_2O)	79.35	(±27.37)	81.76	(±24.52)	88.88	(±22.59)	0.357
**Delayed recovery** (Median)	44	(38.9%)	86	(55.1%)	8	(44.4%)	0.010*
**Delayed recovery** (75th percentile)	17	(15.0%)	47	(30.1%)	4	(22.2%)	0.010*
**In-hospital complications**	20	(17.7%)	47	(30.1%)	4	(22.2%)	0.064
**POAF**	32	(28.3%)	36	(23.1%)	7	(38.9%)	0.279

B) Binary multivariate regression analyses for delayed recovery of physical functioning, and postoperative in-hospital complications stratified in SRT and 2MWT group

	Time to mILAS 0 ≥4 days (median)			Time to mILAS 0 ≥6 days (75th percentile)			Occurrence of ≥1 complication		
	OR	95%	P	OR	95%	P	OR	95%	P
**SRT group (n=113)**									
**SRT WR_{peak}**	0.600	(0.308-1.170)	0.134	0.622	(0.278-1.392)	0.248	0.809	(0.372-1.757)	0.592
**FitMáx VO_{2peak}**	0.879	(0.787-0.981)	0.022*	0.800	(0.652-0.981)	0.033*	0.922	(0.809-1.052)	0.229
**2MWT group (n=156)**									
**2MWT (distance)**	0.992	(0.983-1.002)	0.110	0.990	(0.980-1.000)	0.051	0.992	(0.982-1.001)	0.084
**FitMáx VO_{2peak}**	0.896	(0.833-0.963)	0.003*	0.890	(0.816-0.970)	0.008*	0.910	(0.840-0.986)	0.020*

C) Spearman's Rank-Order Correlation between cardiorespiratory fitness variables		
	Spearman's p	P-value
**FitMáx & SRT**	0.713	<0.001
**FitMáx & 2MWT**	0.592	<0.001

*Data displayed as mean (±standard deviation), median (interquatile range), or number (percentage). A) P-values were obtained using one-way ANOVA, or Chi-square test with post-hoc analyses. Significant difference (0.05> p-value) between groups: * significant difference SRT and 2MWT B) P-values were obtained using Logistic multivariate regression analyses C) P-values were obtained using Spearman's rho (p) correlation.*

Abbreviations: Edmonton frailty scale; HGS, hand grip strength; MIP, maximum inspiratory pressure; POAF, postoperative atrial fibrillation; SRT, Steep Ramp Test; TUG, timed up-and-go; 2MWT, two-minute walk test.
